# The Efficacy of Bethesda System for Prediction of Thyroid Malignancies- A 9 Year Experience from a Tertiary Center 

**DOI:** 10.22038/ijorl.2021.50538.2687

**Published:** 2021-07

**Authors:** Hamdan Ahmed Pasha, Ainulakbar Mughal, Muhammad Wasif, Rahim Dhanani, Syed Arish Haider, Sayed Akbar Abbas

**Affiliations:** 1 *Department of Otolaryngology Head and Neck Surgery, Jinnah Medical College Hospital, Karachi, Pakistan.*; 2 *Department of Otolaryngology Head and Neck Surgery, Aga Khan University Hospital, Karachi, Pakistan.*; 3 *Department of Postgraduate Medical Training, Aga Khan University Hospital, Karachi, Pakistan.*

**Keywords:** Bethesda, Fine needle aspiration, Malignancy risk, Thyroid nodule

## Abstract

**Introduction:**

The best initial investigation for thyroid nodule is fine needle aspiration (FNA). Bethesda System is an international standardized system of reporting thyroid nodules and recommends subsequent management. Every institution should assess the risk of malignancy in each category to avoid unnecessary thyroid surgeries, with this aim we conducted a review at our center to calculate risk of malignancy in each category.

**Materials and Methods:**

Retrospective 9-year (2009–2018) review of thyroid FNAs done at a tertiary care Centre. The FNA was stratified according to The Bethesda System. Histopathology reports of the operated cases were used to evaluate the cytology for diagnostic accuracy.

**Results:**

There were 495 patients who underwent thyroidectomy. The mean age of the cohort was 42.51 +/- 13.2 years and 387 (78.2%) were females. The frequency of Bethesda categories I, II, III, IV, V, and VI were 9.1%, 55.6%, 16.4%, 6.5%, 9.3%, and 3.2% respectively. Malignancy rate in operated thyroid nodules were 37.8%, 8.4%, 33.3%, 50.0%, 89.1%, and 100% for Bethesda categories I to VI, respectively. The sensitivity, specificity, negative predictive value and positive predictive value and their 95% CIs were calculated as 81.30 (73.28 – 87.76%), 77.06 (72.12 – 81.51%), 91.64 (88.3 – 94.1%) and 57.14 (51.79 – 62.33%). The overall diagnostic accuracy was 78.22 (74.12 – 81.95%).

**Conclusions:**

All the Bethesda categories showed greater malignancy risks than other reported studies. Knowledge of local rates of malignancy is important to accurately predict the risk of malignancy even when reported with internationally accepted nomenclature like the Bethesda System.

## Introduction

One-fifth of the general population have a palpable thyroid nodule while upto 70% of the population can have thyroid nodules on ultrasound (1). It is more commonly found in women than men (2). The assessments of these swellings are mainly concerned around differentiating benign from malignant ones to avoid unnecessary surgery. Fine needle aspiration cytology is simple and affordable to assess thyroid nodules and is now considered as first line diagnostic tool (3). 

The sensitivity and specificity of fine needle aspiration (FNA) ranges from 55% to 98% and 73-100% according to various published international data (4-7). Nevertheless, fine needle aspiration cytology (FNAC) had its limitations. These difficulties depend on the adequacy of the sample, the technique and expertise during the aspiration and analysis of sample and along with the microscopic similarities between benign and malignant follicular neoplasms (8,9). Initially, FNAC reports by different cytopathologists varied in their terminologies and diagnostic criteria that led to confusion in interpretation. In 2007 The National Cancer Institute, Bethesda, Maryland, United States published guidelines to standardize the nomenclature used for the interpretation of FNAs called The Bethesda system for reporting thyroid cytopathology (10). According to this system any thyroid nodule would be initially reported under than one of the six main categories: Non-diagnostic/unsatisfactory, benign, atypical follicular lesion of undetermined significance (AFLUS), “suspicious” for follicular neoplasm (SFN), suspicious for malignancy (SM), and malignant (11). Each subsequent category has an escalating risk of malignancy and respective management guidelines. However, risks of malignancy has varied over multiple studies which led to recommendations regarding institutional rates of malignancy. 

A recent meta-analysis showed that even using ultrasound guidance during FNA, the diagnostic accuracy merits limited confidence due to bias, imprecision and inconsistency (12). Our study reports the malignancy risks in thyroid nodules as per Bethesda categorization by studying the incidence of malignancy in each category. We also compared the accuracy of FNA when done using ultrasound guidance versus direct FNA by palpation of nodule.

## Materials and Methods

We did a retrospective review of nine years in the Department of Otolaryngology & Head and Neck Surgery at Aga Khan University Hospital which is a tertiary care centre in Karachi, the largest city of Pakistan, and gets referrals from all over the country. After Ethical review committee exemption, data was collected from patient’s medical records dated December 2009 to October 2018 on a standard template. We classified FNAs using the Bethesda system introduced in 2007. In November 2018 our centre adopted the 2017 updated Bethesda classification. We used the previous classification to keep our results comparable to other studies. All patients who underwent fine needle aspirations of the thyroid gland were included in the study. Patients who did not undergo surgical resection and those with repeat aspirations were excluded. 1187 nodules were aspirated in the study period. 692 patients did not have surgical intervention and thus no histology was available. After exclusion of these, 495 thyroid nodules were evaluated for presence of malignancy. Data was recorded regarding patient age and gender, FNAC with or without ultrasound guidance, category of Bethesda on fine needle aspiration, surgical intervention done and final histopathology diagnosis. Ultrasound guided aspiration was performed at the discretion of the cytologist. Large palpable nodules underwent direct FNA without the aid of ultrasound. Only category II (benign) was considered negative on FNA and the rest were considered to be indicative of some malignancy and thus positive. True positives/negatives and false positives/negatives were recorded by comparing with malignancy on final histology. Bethesda I (non-diagnostic) category was excluded from the analysis. Cytology was correlated with histology to calculate sensitivity, specificity, positive and negative predictive values using standard formulas for these. Results were stratified on the basis of ultrasound guidance used during FNA. All Statistical analysis was done using software SPSS version 23. Students T test and Chi square tests were applied where appropriate. *P*≤0.05 was considered statistically significant.

## Results

1187 fine needle aspirations were done at a tertiary care center in the nine year time period. 495 patients underwent surgical excision and their histology was recorded. The mean age of the cohort was 42.51 +/- 13.2 years. There were 387 females (78.2%) and 108 (21.8%) males. The youngest patient was 9 year old female and the eldest was an 85 year old male. 

The overall rate of malignant nodules was 140 out of 495 (28.3%). 

Patients under the age of 55 years had a malignancy rate of 27.8% while those above 55 years had 30.9% (P-value 0.573).Ultrasound guided FNA was performed in 388 (78.4%) patients. The frequency of non-diagnostic, benign, atypia of undetermined significance (AUS), follicular neoplasm, suspected for malignancy, and malignant cases was 9.1%, 55.6%, 16.4%, 6.5%, 9.3%, and 3.2% respectively. No statistical significance was found in the rate of non-diagnostic results between non-ultrasound guided FNA versus ultrasound-guided FNA (T-test) (9.3% vs. 9.7%, p-value 0.917). Malignancy rate in operated thyroid nodules were 37.8%, 8.4%, 33.3%, 50.0%, 89.1%, and 100% for Bethesda categories I to VI, respectively (Table 1). After excluding the 45 non-diagnostic FNAs we calculated the true and false positives and negatives for the remaining 450 aspirates. Only category II (benign) was considered negative on FNA and the rest were considered to be indicative of some malignancy and thus positive. 

**Table 1:** Risk of Malignancy (ROM) in each category of Bethesda system compared with other series

**TBSRTC Category on FNA**	**ROM in our study**	**Cibas et al. (2009)** ^1^ ** (Estimated)**	**Bongiovanni ** **et al. (2012)** ^2^ ** (N=6362)**	**Sheffield et al. (2014)** ^3^ **(N=8044)**	**Krauss et al. (2016)** ^4^ **(N=8214)**
Non diagnostic (n=45)	37.8%	1 – 4%	16.8%	18.7%	12%
Benign (n=275)	8.4%	0 – 3%	3.7%	6.5%	5%
Atypia of undetermined significance (n=81)	33.3%	5 – 15%	15.9%	28.3%	17%
Follicular neoplasm (n=32)	50.0%	15 – 30%	26.1%	33.1%	25%
Suspicion of Malignancy (n=46)	89.1%	60 – 75%	75.5%	65%	72%
Malignant (n=16)	100%	97 – 99%	98.6%	98.6%	98%

^1 ^Cibas ES, et al. Am J Clin Pathol 2009;132:658–65.

^ 2 ^Bongiovanni M, et al. Acta Cytol 2012;56:333–9.

^3 ^Sheffield BS, et al. Expert Rev Endocrinol Metab 2014;9:97–110.

^ 4 ^Krauss EA, et al. Arch Pathol Lab Med 2016;140:1121–31.

The most common final histologic diagnoses upon resection was found to be benign adenomas in 334 (67.5%). Papillary carcinoma was the most common malignancy accounting for 104 (21.0%) followed by follicular carcinoma in 18 (3.6%) and medullary carcinoma in 7 (1.4%). Two cases each of anaplastic carcinoma and lymphomas were diagnosed on final histology.

 The remaining 28 (5.6%) cases showed either thyroiditis or rare entities namely insular carcinomas squamous cell carcinoma, lymphoepithelial cyst, tuberculosis, malignant peripheral nerve sheath tumor, low grade sclerosing mucoepidermoid carcinoma, Hurthle cell Carcinoma and sarcoma (Table.2).

**Table 2:** Histopathological diagnosis as per different categories

**Category**	**Frequency (%)**
*Benign*	*334 (67.5%)*
Adenoma	*334 (67.5%)*
*Malignant*	*133 (26.9%)*
Papillary carcinoma	*104 (21%)*
Follicular carcinoma	*18 (3.6%)*
Medullary carcinoma	*7 (1.4%)*
Anaplastic carcinoma	*2*
Lymphoma	*2*
*Other*	*28 (5.6%)*
Thyroiditis	*18*
Insular carcinoma	*3*
Squamous cell carcinoma	*1*
Lymphoepithelial cyst	*1*
Tuberculosis	*1*
Malignant peripheral nerve sheath tumor	*1*
Low grade sclerosing mucoepidermoid carcinoma	*1*
Hurthle cell carcinoma	*1*
Sarcoma	*1*

Overall, the sensitivity, specificity, negative predictive value and positive predictive value and their 95% CIs were calculated as 81.30 (73.28 – 87.76%), 77.06 (72.12 – 81.51%), 91.64 (88.3 – 94.1%) and 57.14 (51.79 – 62.33%). The overall diagnostic accuracy was 78.22 (74.12 – 81.95%). We calculated the sensitivity, specificity, negative predictive value, positive predictive value and diagnostic accuracy by stratifying on the basis of ultrasound guidance. T-Test showed that the values calculated did not differ significantly on analysis (Table 3). 

**Table 3:** Diagnostic Accuracy of FNA with or without Ultrasound guidance

**Category**	**US aided**	**Without US**	**Overall**
Sensitivity	81.63%	80.00%	81.30 (73.28 – 87.76%)
Specificity	75.29%	83.30%	77.06 (72.12 – 81.51%)
Positive Predictive Value	55.94%	62.50%	57.14 (51.79 – 62.33%)
Negative Predictive Value	91.43%	92.31%	91.64 (88.30 – 94.10%)
Diagnostic Accuracy	77.05%	82.47%	78.22 (74.12 – 81.95%)
			

## Discussion

The Bethesda System for Reporting Thyroid Cytopathology (TBSRTC) implies risks of malignancy in each category (13, 14). However the acquisition and interpretation of thyroid nodule aspirates are very subjective and prone to variability (15,16). It is thus recommended for each institution to have their internal audits as a quality control with TBSRTC as the benchmark for more accurate patient counselling prior to surgery. (17) It also contributes to the existing data and helps ascertain actual, often greater risks, rather than the earliest estimations by Cibas (18). 

The sensitivity of 81% and overall accuracy of 78.2% makes FNA a good initial diagnostic modality and a negative predictive value of 91.6% helps rule out malignancy. The meta-analysis by Baloch et al found a higher sensitivity of 97% and higher negative predictive value of 96.3% with a lower overall diagnostic accuracy of 68.8% (14). The addition of ultrasound guidance during FNA did not impact the diagnostic probabilities or the overall diagnostic accuracy in our study. However, the implied risk of malignancy for each category is necessary while counselling patients for subsequent clinical management.

Cibas suggested that non-diagnostic FNAs should not make more than 10% of all the FNAs done and this was seen at our center too where non diagnostic aspirates made up 9.1% of almost 500 samples. The risk of malignancy (ROM) in non-diagnostic aspirates was very high in our study (37.8%). Although the initially suggested estimated risk was of 1-4% in this category a recent meta-analysis reported a risk of malignancy of 16.8% (19). The number of non-diagnostic samples (45) was small in our study however the frequency of malignant nodules was significant. The greater frequency of malignancy in Bethesda I could be due to inexperienced sampling causing poor cellularity and /or technical difficulty. Our findings are supported by others from Asian region who have reported malignancy rates from 40 – 70% (20-22). We did not find the aid of ultrasound guidance to reduce the number of non-diagnostic aspirates either. Few authors suggest that the time interval between aspirates following non-diagnostic should be of at least 3 months while others have reported no difference in the diagnostic yield (23). In our study repeat aspirations was not done in any non-diagnostic FNA and these aspirates were excluded during the calculation of sensitivity and specificity.

A total of 275 (55.6%) aspirates were Bethesda II (benign) in our study. 23 (8.4%) were malignant. On reviewing such a high risk of malignancy we found that there were 9 cases of papillary micro carcinoma in our study. After removing these cases of false negatives the risk of malignancy came down to 14 out of 275 (5.1%) which was closer to recommended risk in literature. Micro carcinomas increasing the risk of malignancy in Bethesda II nodules was also seen by Wu et al (24). 

The use of AUS should not be more than 7% and should be reserved as the last resort when no other confirmative diagnosis can be made on cytology (18). AUS was more frequently used making up to 16.4% of the FNAs and subsequently showed a much higher risk of malignancy of 33% as compared to 5 – 15% as estimated by Cibas or 17% as per a recent meta-analysis (19). This greater use of AUS category and greater malignancy has been reported by others as well (25). Many factors could have contributed to this like insufficient sampling, sampling from indeterminate part of the nodule and cytologist interpretation. However, greater frequency of malignancies in all categories of the FNAs as seen in our study might indicate lack of confidence during cytological diagnosis and preference to use the indeterminate categories. 

Recently, further division of AUS / FLUS based on presence of nuclear atypia has been suggested (26). Nuclear atypia is considered to be more predictive of malignancy in these indeterminate categories. Also, newer molecular testing has been used as an adjunct in these aspirates but currently are not widely available across the world nor at our center (27,28). 

Most of the studies quoted here are from pre-noninvasive follicular thyroid neoplasm with papillary-like nuclear features (NIFTP) era and at our institute also NIFTP diagnosis was not found in any of the final histology cases. Hence, its impact on the risk of malignancy could not be estimated. However if included as a benign pathology it might have reduced the risk of malignancy especially for Bethesda III although Asian literature has shown to report fewer cases of NIFTP (29,30). 

We report a higher risk of malignancy in all the other Bethesda categories also. For Bethesda IV, V and VI the risk was 50.0%, 89.1%, and 100% respectively. This could indicate the higher prevalence of carcinoma in thyroid nodules found in our region. It is possible that being a tertiary referral center we treated patients who had more suspicious nodules which gives rise to inherent selection bias. Other authors have reported similar results. Kim et al reported similar multicenter data from Korea and suggested to revisit the guidelines of TBSRTC (31). Another possible reason for high ROM in Bethesda V would be lack of confident interpretation by readers where a malignant smear would be read as only suspicious for malignancy and only the frankly malignant smears were labelled as category VI giving risk of malignancy to be 100%.

We searched for malignancy rates reported from our country to control the heterogeneity of the populations being compared. We found only one study that reported malignancy risks on 61 nodules (32). Although their sample size was small, they similarly reported malignancy risk of 11.1% in their 45 benign nodules and 33.3% in six Bethesda III category nodules. All six nodules of Bethesda V and VI category were found to be malignant in their study.

Our study has its limitations. First, taking into account only operated cases has its unavoidable selection bias. We also did not look into patients who had repeat FNAs as this could have interfered with the classification of cytology to be considered false positive or false negative. Patients who opted for surgery in such a scenario could have had suspicious radiology or clinical findings. Also, the cytology technique either alcohol or liquid based could have an impact on diagnostic ability of FNA or this was not considered in our study. 

## Conclusion

We report a much higher risk of malignancy as compared to the contemporary western literature. TBSRTC is a reliable initial investigation tool for thyroid nodules but careful interpretation of the aspirate is imperative to predict the risk of malignancy. There are pitfalls and variations in the diagnosis of indeterminate nodules and refined criteria to place each aspirate into a defined category will be helpful. We recommend that every institution should audit their frequencies and malignancy rates to serve as a quality indicator during patient care. 
